# Periovulatory follicular fluid levels of estradiol trigger inflammatory and DNA damage responses in oviduct epithelial cells

**DOI:** 10.1371/journal.pone.0172192

**Published:** 2017-02-23

**Authors:** Sergio E. Palma-Vera, Jennifer Schoen, Shuai Chen

**Affiliations:** 1 Institute of Reproductive Biology, Leibniz Institute for Farm Animal Biology (FBN), Dummerstorf, Germany; 2 Institute of Veterinary Biochemistry, Department of Veterinary Medicine, Freie Universitaet Berlin, Berlin, Germany; 3 College of Life Science, Hebei University, Baoding, People’s Republic of China; Universite du Quebec a Trois-Rivieres, CANADA

## Abstract

**Objective:**

Ovarian steroid hormones (mainly E2 and P4) regulate oviduct physiology. Serum-E2 acts on the oviduct epithelium from the basolateral cell compartment. Upon ovulation, the apical compartment of the oviduct epithelium is temporarily exposed to follicular fluid, which contains much higher levels of E2 than serum. The aim of this study was to evaluate the effects of human periovulatory follicular fluid levels of E2 on oviduct epithelial cells using two porcine *in vitro* models.

**Methods:**

A cell line derived from the porcine oviductal epithelium (CCLV-RIE270) was characterized (lineage markers, proliferation characteristics and transformation status). Primary porcine oviduct epithelial cells (POEC) were cultured in air-liquid interface and differentiation was assessed histologically. Both cultures were exposed to E2 (10 ng/ml and 200 ng/ml). Proliferation of CCLV-RIE270 and POEC was determined by real-time impedance monitoring and immunohistochemical detection of Ki67. Furthermore, marker gene expression for DNA damage response (DDR) and inflammation was quantified.

**Results:**

CCLV-RIE270 was not transformed and exhibited properties of secretory oviduct epithelial cells. Periovulatory follicular fluid levels of E2 (200 ng/ml) upregulated the expression of inflammatory genes in CCLV-RIE270 but not in POEC (except for *IL8*). Expression of DDR genes was elevated in both models. A significant increase in cell proliferation could not be detected in response to E2.

**Conclusions:**

CCLV-RIE270 and POEC are complementary models to evaluate the consequences of oviduct exposure to follicular fluid components. Single administration of periovulatory follicular fluid E2 levels trigger inflammatory and DNA damage responses, but not proliferation in oviduct epithelial cells.

## Introduction

Within the estrous cycle, changing serum levels of ovarian steroid hormones (mainly estradiol, E2, and progesterone, P4) regulate oviduct physiology in mammals [1–4], including transport and maturation of oocytes, sperm and early embryo development [5]. During these cyclic changes, serum-E2 acts on the oviduct epithelium from the basolateral cell compartment. Upon ovulation, the luminal side (apical compartment) of the oviduct epithelium is temporarily exposed to follicular fluid, which induces an inflammatory-like process with macrophage infiltration and enhanced DNA damage [6].

Follicular fluid contains much higher levels of E2 [7] than serum (up to 200 ng/ml). The impact of such high concentrations of apical E2 on the oviduct epithelium has not yet been elucidated. However, a recent study showed that already 10 nM E2 (~2.72 ng/ml) could upregulate transcription of the inflammatory chemokine IL8 in human oviductal epithelial cells *in vitro* [8].

In other cell types it was reported that E2 can have genotoxic and proliferative effects. Genotoxicity results from accumulation of ROS and depurinating adducts after oxidative metabolism of E2, whereas its proliferative properties result from the interaction of E2 with its nuclear and membrane-bound receptors [9,10]. Also, it was shown that E2 can either promote or inhibit inflammation, determined by the cell type and E2 concentrations among other factors [11].

Pig is an alternative model species for biomedical research, allowing sample collection at large scales for the establishment of complex *in vitro* models [12,13]. Recently, our group has established an air-liquid-interphase (ALI) model for culturing primary porcine oviduct epithelial cells (POEC) [14,15]. These cells become polarized, exhibit ciliated and secretory phenotypes and are able to respond to basolateral E2 and P4, thus, preserving the native features of the oviductal epithelium during the estrus cycle.

In the present study, we aim to evaluate the effects of apical administration of human periovulatory follicular levels of E2. We employed two porcine cell models: a) an oviductal secretory cell line (CCLV-RIE270) and b) a polarized and *in vivo*-like POEC culture system that allows hormonal manipulation from both the basolateral and apical compartment [14–17]. We hypothesize that human periovulatory follicular levels of E2 (as they are found in the follicular fluid during ovulation) triggers DNA damage response (DDR), proliferation and inflammatory response simultaneously.

## Materials and methods

Research material for this study derived from two sources: 1) porcine oviducts collected at a commercial slaughterhouse (Teterower Fleisch GmbH, Koppelbergstraße 2, 17166 Teterow, Germany) from pigs slaughtered for the purpose of meat production and 2) a porcine cell line (CCLV-RIE270) kindly provided by the cell line collection of the Friedrich-Loeffler-Institute, Germany. Thus, animals were not specifically killed for the purpose of this study and ethical approval was not required.

### Reagents

Phenol red free DMEM, accutase and GlutaMax were products of Gibco (Thermo Fisher Scientific, Dreieich, Germany). Reduced glutathione, collagenase 1A, bovine serum albumin (BSA), ascorbic acid, E2 and primers for qPCR were provided by Sigma-Aldrich (Neustadt, Germany). Other cell culture reagents and additives were purchased from Biochrom (division of Merck Millipore, Berlin, Germany) unless otherwise specified. Reagents used for RT-qPCR were obtained from Thermo Fisher Scientific (Dreieich, Germany).

### Characterization of CCLV-RIE270 cells

The CCLV-RIE270 cells (1x10^6^) were plated in T-25 cell culture flasks with 8 ml culture medium: DMEM (without phenol red) supplemented with 10% fetal bovine serum, 4 mM GlutaMax and 1 mM sodium pyruvate. Cells were passaged every 3 to 4 days at the ratio of 1:3 once cells reached 80% confluence.

#### Transformation status

We evaluated anchorage independent growth via soft agar colony formation by the CytoSelect™ 96-Well Cell Transformation Assay kit (Cell Biolabs, CA, USA) following the manufacturer’s instructions. A transformed human embryonal kidney cell line (HEK-293) was used as positive control, while a mouse fibroblast cell line (NIH-3T3) was taken as negative control. Briefly, Cells (5×10^3^/well) were seeded in triplicate onto a 96-well plate. After 7 days incubation at 37°C and 5% CO_2_, cells were lysed and labeled with CyQuant GRdye. Fluorescence was determined with CytoFluor II Microplate Reader (Thermo Fisher Scientific, Dreieich, Germany) using a 485/530 nm filter set.

#### Proliferation characteristics

To check how cell growth and proliferation are affected by the seeding density, we performed impedance monitoring using the xCelligence system (Roche, Mannheim, Germany) following the manufacturer’s instructions. This system monitors cell proliferation in real-time by measuring electrical resistance across electrodes on the bottom of a 96-well tissue culture plate (E-Plate). Cells were seeded at the density of 5x10^3^, 1x10^4^, 2x10^4^ and 4x10^4^/well. For each seeding density, 5 replicates were included. Electrical impedance was monitored at intervals of 15 min. Cell impedance was represented as normalized cell index.

#### Detection of lineage markers

Markers for secretory oviduct epithelial cells (OVGP1, PAX-8, ESR1) and epithelial markers (Cytokeratin, ß-Catenin) were evaluated by immunofluorescence (IF). Cells were grown on glass cover slips, fixed with histofix 4% (Carl Roth, Karlsruhe, Germany) overnight at 4°C, and unspecific binding sites were blocked with either 5% BSA plus 10% goat serum (Abcam, Cambridge, UK) or Roti-ImmunoBlock (1:50, Carl Roth, Karlsruhe, Germany) for 1 h at room temperature. Cells were incubated with the primary antibody overnight at 4°C. Primary antibodies and their respective dilutions (in blocking buffer) are listed in Table 1. Corresponding secondary antibodies were purchased from Thermo Fisher Scientific (Dreieich, Germany) and diluted in PBS + 1% BSA: goat anti rabbit Alexa 647 (1:200, A21245), donkey anti goat Alex 568 (1:40, A11057), and goat anti mouse Alexa 488 (1:40, A11017). Incubation time was 1 h at room temperature. Nuclei were counterstained either with TO-PRO-3 iodide (Mobitec, Berkheim, Germany) or SYBR Green I (Mobitec, Berkheim, Germany). Images were captured by the confocal laser scanning microscope LSM 800 equipped with ZEN software (Carl Zeiss, Oberkochen, Germany).

**Table 1:** List of primary antibodies applied for IF / IHC.

Application	Antigen	Dilution	Blocking	Supplier	Cat. number
IF	β-Catenin	1:200	BSA / GS	Cell Signaling	9562
IF	ESR1	1:50	BSA / GS	Santa Cruz	C-311
IF	PAX-8	1:100	BSA / GS	Proteintech	21383-1-AP
IF	OVGP1	1:100	RB	Santa Cruz	SC-46432
IF	Acetylated Tubulin	1:1000	BSA / GS	Sigma	T7451-100UL
IF	Cytokeratin	1:100	BSA / GS	Dako	Clone AE1/AE3
IHC	Ki67	ready to use	BSA / GS	BioLogo	KI505

BSA / GS = 5% BSA + 10% goat serum, RB = Roti-Immunoblock.

### Primary POEC

After collection at the slaughterhouse, oviduct tissues were immediately transported on ice to the laboratory. Surrounding tissue was trimmed and oviducts were washed in PBS. Oviduct epithelial cells were isolated as previously described [14]. Briefly, oviducts were filled with collagenase and squeezed to detach epithelial clusters. Cell clusters were further singled out by accutase and either seeded immediately or stored in liquid nitrogen for later use.

Cells were seeded on 12-well hanging inserts (PET membrane, 0.4 μm pore size, Merck Millipore, Darmstadt, Germany) at a concentration of 4.5×10^5^/400 μl of growth medium. Inserts were placed into 12-well plates containing 1.5 ml of growth medium consisting of two parts of phenol red free Ham’s F12 (with 10% fetal bovine serum), one part of 3T3 conditioned medium (produced as described previously [14]), 100 U/ml penicillin, 100 μg/ml streptomycin, 50 μg/ml gentamicin, 1 μg/ml amphotericin B, 10 μg/ml reduced glutathione and 10 μg/ml ascorbic acid. Cells were incubated in a humid chamber at 37°C with 5% CO_2_. After 3 days, the medium inside the insert was removed, allowing cells to remain in an ALI. Medium changes were performed twice a week and cultures were treated with E2 after 3 weeks of culture.

#### Characterization of POEC

After 3 weeks of cultivation, membranes were cut out of the inserts, sequentially fixed with Bouin´s solution and histofix 4%, dehydrated in a graded ethanol series, and then vertically embedded in paraplast (Leica, Wetzlar, Germany) as described previously [15]. Sections of 4 μm were prepared for hematoxylin-eosin staining or immunohistochemical (IHC) detection.

The presence of ciliary marker Acetylated Tubulin was detected by IF. To illustrate the cilia structure from an aerial view, the membrane was removed from the insert, fixed in 4% histofix, and processed directly for staining without embedding in paraplast. Protocol for IF was performed as described in the paragraph “Detection of lineage markers”.

### E2 stimulation experiments

Two different concentrations of E2 (10 ng/ml and 200 ng/ml) were employed for stimulation, as E2 levels reported for human follicular fluid around the time point of ovulation vary widely in the literature [18,19]. CCLV-RIE270 (passage 32, 5 replicates) was stimulated with E2 in a total volume of 8 ml of medium for 20 min, 3 h and 24 h in T-25 flasks.

After 3 weeks ALI culture, POEC from 5 different animals were apically exposed to E2 in a total volume of 400 μl for 3 h and 24 h. The amount of E2 per surface unit (cm^2^) was taken into account, in order to expose CCLV-RIE270 and POEC to comparable amounts of E2. As controls, only vehicle (ethanol) was applied to cultures.

### Proliferation assays

After E2 stimulation, proliferation of CCLV-RIE270 was measured by real-time impedance monitoring using the xCelligence system. Cells were seeded onto the E-Plate at a density of 5x10^3^/well. After 48 h of culture, cells were treated with E2 (10 ng/ml, 200 ng/ml and ethanol as negative control) for 24 h (5 replicates). Thereafter, medium was refreshed, and cell growth was further monitored up to 200 h.

Furthermore, proliferation of CCLV-RIE270 and POEC stimulated with E2 for 24 h was assessed by IHC detection of Ki67. CCLV-RIE270 grown on glass cover slips (n = 5 replicates per group) were treated with E2 (10 or 200ng/ml) or solvent for 24h when they reached ~50% confluence and fixed in ice-cold methanol. Cells were washed, blocked and subjected to incubation with anti-Ki67 antibody at 4°C overnight. Antigen detection was visualized by the EnVision Dual Link System-HRP kit (Dako, Hamburg, Germany) and counterstained with hemalum. Cover slips were mounted on glass slides and at least 13 consecutive microscopic pictures were taken (magnification x400). Images were acquired with an Axio Imager A1 upright microscope equipped with AxioVison Rel.4.8 software (Carl Zeiss, Oberkochen, Germany). The total number of nuclei and Ki67 positive cells were counted using Image J software. POEC membranes were embedded in paraplast as described above, sections of approx. 4 μm were subjected to heat-induced antigen retrieval in citrate buffer (10 mM, pH 6.0, 3 min), followed by incubation with anti-Ki67 antibody at 4°C overnight. Staining and acquisition of micrographs (at least 5 pictures per sample) was performed as described above. The total number of nuclei and Ki67 positive cells over the membrane length were counted for all samples (n = 5 animals).

### Gene expression analysis

Gene expression of CCLV-RIE270 and POEC was assessed by RT-qPCR. Primer sequences, product sizes and transcript identifiers are shown in Table 2. Annealing temperature was 60°C for all primers.

**Table 2:** Primer sequences used for RT-qPCR.

Gene	Primer sequence (5′- 3′)	Amplicon (bp)	Accession number
GAPDH	F: ATTCCACCCACGGCAAGTTC	225	NM_001206359.1
	R: AAGGGGCAGAGATGATGACC		
ACTB	F: CAACTGGGACGACATGGAG	234	XM_003124280.4
	R: GAGTCCATCACGATGCCAG		
SDHA	F: CTACAAGGGGCAGGTTCTGA	141	DQ845177.1
	R: AAGACAACGAGGTCCAGGAG		
ESR1	F: AGGGAAGCTCCTGTTTGCTCC	234	NM_214220.1
	R:CGGTGGATATGGTCCTTCTCT		
PGR	F: TGAGAGCACTAGATGCCGTTGCT	197	NM_001166488.1
	R: AGAACTCGAAGTGTCGGGTTTGGT		
IL8	F: GCTCTCTGTGAGGCTGCAGTT	62	XM_003361958.3
	R: TTTATGCACTGGCATCGAAGTT		
IL6	F: ATAAGGGAAATGTCGAGGCTG	88	NM_001252429.1
	R: GTGGCTTTGTCTGGATTCTTTC		
PTGS2	F: AGAGCTTCCCGATTCAAAGG	144	NM_214321.1
	R: CCTCGCTTCTGATCTGTCTTG		
CAT	F: TCAGGACAATCAAGGTGGGGCT	81	NM_214301.2
	R: TGTTCCAAGGCCGAATGCGT		
C3	F:AACAAGGGCAAGCTGTTGAAGGTG	119	NM_214009.1
	R:TAATAAGCCACCAGGCGGAAGGAA		
CDKN1A	F: AGAGTCGGTAGTTGGGAGATC	87	XM_001929558.3
	R: CTCTGACATGGTGCCTGTG		
DDB2	F: GATTCGGGTTTACTCTGCCTC	150	XM_013994354.1
	R: AAATTAGGATCTGGGTATCGGC		
GADD45G	F: ACTCTGGAAGAAGTTCGCGG	164	NM_001185129.1
	R: TTGTCGGGGTCCACATTCAG		
TP53	F: GGAACAGCTTTGAGGTGCGTGTTT	182	NM_213824.3
	R: ATACTCGCCATCCAGTGGCTTCTT		
BAX	F: GCTGACGGCAACTTCAACTG	202	XM_005664710.2
	R: GCGTCCCAAAGTAGGAGAGG		

Cultures were washed with PBS before RNA was extracted with a commercial kit following the manufacturer’s instructions (NucleoSpin RNA, Macherey-Nagel, Dueren, Germany). RNA quantity and purity were measured by NanoDrop ND-1000 (Peqlab Biotechnology, Erlangen, Germany), whereas RNA quality was measured by Agilent 2100 Bioanalyzer (Agilent Technologies, Waldbronn, Germany). All samples used in this study exhibited an RNA integrity number higher than 9.

RNA (1μg) was treated with DNase I (1 U, 30 min at 37°C), linearized at 65°C for 5 min and annealed to a mixture (1:1 v/v) of oligo dT and random hexamers (2.5 μM each). Then, RNA was converted into cDNA by adding dNTPs (0.5 mM) and RevertAid reverse transcriptase (200 U, 25°C for 10 min, 42°C for 60 min and 70°C for 10 min) in a total volume of 20 μL. Quantitative PCR was performed in duplicates using LightCycler 96 system (Roche, Mannheim, Germany). For this, cDNA was amplified in a final volume of 12 μl containing 0.5 μM forward primer, 0.5 μM reverse primer, 1X FastStrat Essential DNA Green Master (Roche, Mannheim, Germany). PCR amplification was performed as follows: initial denaturation at 95°C for 10 min, 45 cycles of 95°C for 20 s, 60°C for 20 s, and 72°C for 10 s.

Expression levels of mRNAs were determined in duplicate and relative gene expression was calculated by applying the 2^-ΔΔCT^ method [20], corrected for PCR efficiency. Reference genes for normalization were determined using the GeNorm algorithm, part of the R package “NormqPCR” [21].

### Statistical analysis

#### POEC

Data from POEC was tested for normality (Shapiro Wilk test). Normal data was analyzed by repeated-measures analysis of variance (ANOVA), followed by pairwise paired t-test with Bonferroni correction. Data that did not follow a normal distribution was compared using Friedman rank sum test, followed by pairwise paired Wilcoxon rank sum test with Bonferroni correction. For each experiment, 5 animals were used. All analyses were performed using the R statistical software.

#### CCLV-RIE270

Data from CCLV-RIE270 was tested for normality (Shapiro Wilk test). Normal data was analyzed by one way ANOVA, followed by pairwise t-test with Bonferroni correction. Data that did not follow a normal distribution was compared using Kruskal-Wallis rank sum test, followed by pairwise Wilcoxon rank sum test with Bonferroni correction. For each experiment, 5 biological replicates were used. All analyses were performed using the R statistical software.

## Results

### Characterization of CCLV-RIE270 and POEC

**Fig 1:**
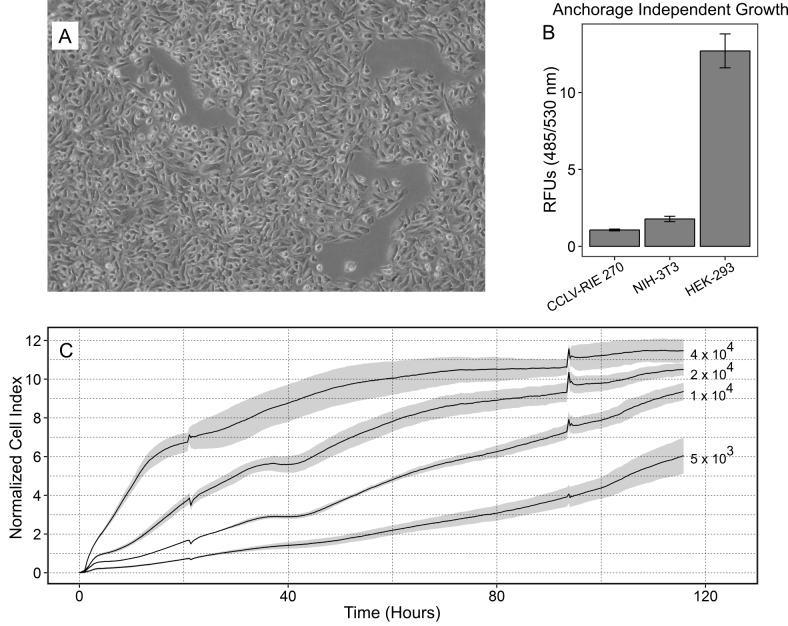
Characterization of CCLV-RIE270. A: Phase-contrast micrograph, magnification x100; B: Anchorage independent growth: comparison among CCLV-RIE270, NIH-3T3 and HEK-293 cell lines. RFUs, relative fluorescence units; n = 3 replicates. C: Proliferation curves of CCLV-RIE270 seeded at different densities as measured by real-time impedance monitoring (xCelligence), shaded regions indicate standard deviations (n = 5 replicates).

CCLV-RIE270 exhibited typical epithelial arrangement: polygonal shape and growth in discrete islands (Fig 1A). Cells did not show proliferation in soft agar and were thus not transformed (Fig 1B). The proliferative pattern of CCLV-RIE270 was evaluated by impedance measurement. Four seeding densities (5x10^3^, 1x10^4^, 2x10^4^ and 4x10^4^/well) were tested (Fig 1C). Generally, when seeded at 1-2x10^4^cells/well, 80% confluence was reached within 3–5 days. For the purpose of defining the most suitable seeding number to evaluate the effect of E2 on proliferation, an optimal growth curve would reach its plateau (confluence) towards the endpoint of the experiment (approx. 200 h), leaving room to evaluate the effects of E2 stimulation starting after 48 h. According to this criterion, the lowest seeding amount (5x10^3^cells/well) was chosen for E2 stimulation.

**Fig 2:**
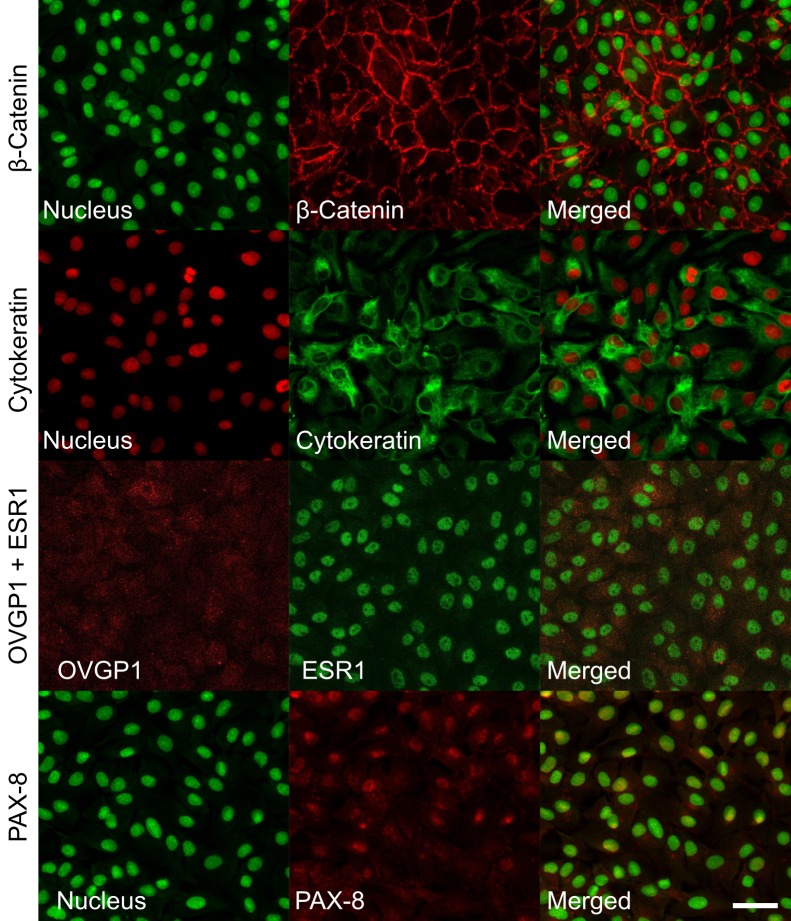
Markers for oviduct secretory cells in CCLV-RIE270. Epithelial markers (β-Catenin, Cytokeratin) and lineage markers (OVGP1, ESR1 and PAX-8) detected by immunofluorescence. Scale bar = 40 μm.

Immunofluorescence demonstrated that CCLV-RIE270 express lineage markers for oviductal secretory cells (OVGP1, PAX-8 and ESR1) as well as epithelial markers (Cytokeratin, ß-Catenin), validating the secretory origin of CCLV-RIE270 (Fig 2).

**Fig 3:**
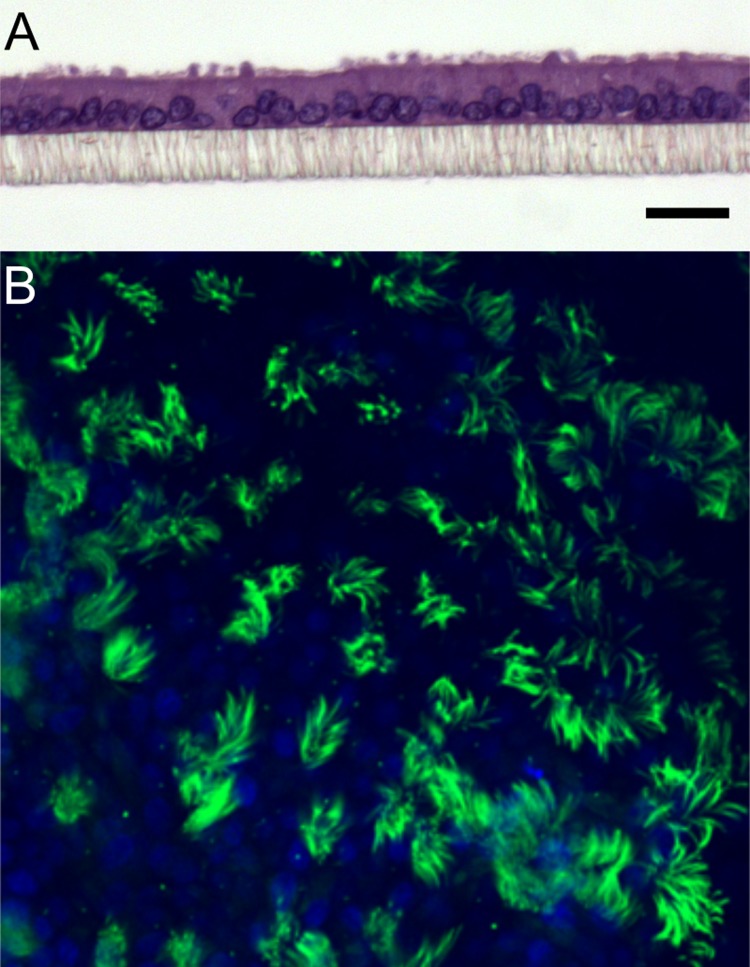
Characterization of POEC after 3 weeks of ALI culture. A: Representative cross-section of paraffin embedded culture stained with hematoxylin-eosin, Scale bar = 20 μm; B: Immunofluorescent staining of cilia (Acetylated Tubulin, green). Nuclei (blue) were labeled with DAPI.

POEC exhibited epithelial polarity and both ciliated and secretory phenotypes after 3 weeks of culture (Fig 3A). Presence of ciliated cells was visualized by the ciliary marker Acetylated Tubulin (Fig 3B).

### Effect of E2 on proliferation in oviduct epithelial cells

**Fig 4:**
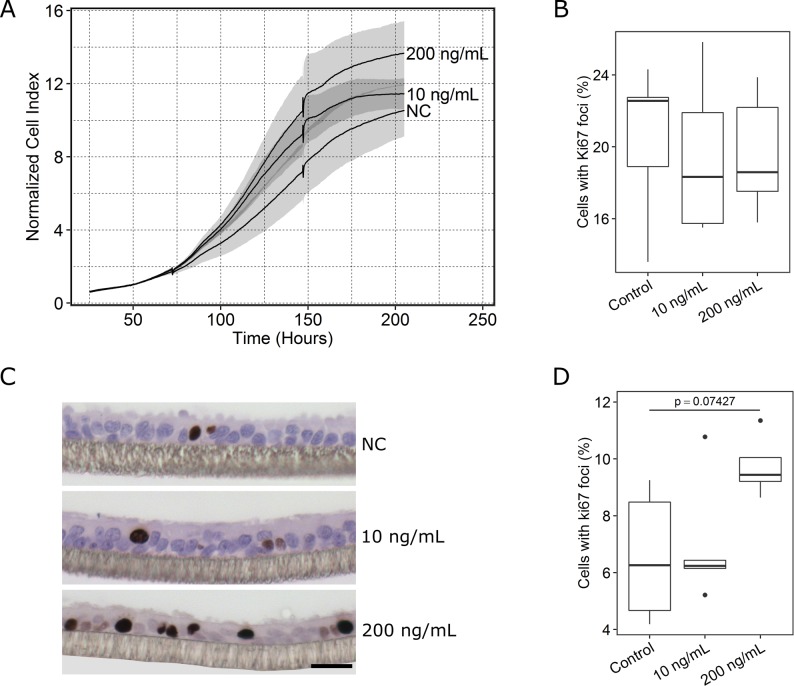
Cell proliferation in response to periovulatory follicular concentrations of E2 in CCLV-RIE270 and POEC. A: After 2 days propagation, CCLV-RIE270 cells were treated with E2 (10 ng/ml, 200 ng/ml) or vehicle (ethanol) for 24 h and then further cultured up to 200 h. Cell growth was monitored in real-time using the xCelligence system, shaded regions indicate standard deviations (n = 5 replicates); B: Percentage of Ki67 positive CCLV-RIE270 cells after 24 h E2 stimulation, n = 5 animals; C: representative images for Ki67 staining in POEC. Scale bar = 20 μm. NC, negative control; D: Percentage of Ki67 positive POEC after 24 h E2 stimulation, n = 5 animals.

After defining the appropriate seeding density (5x10^3^/well), proliferation of CCLV-RIE270 in response to E2 was evaluated by impedance monitoring. Visual inspection of Fig 4A suggests a dose dependent shift in the growth curve of CCLV-RIE270. However, the differences between the groups do not reach statistical significance.

We also determined the percentage of positive Ki67 foci to assess proliferation of CCLV-RIE270 (Fig 4B) and POEC (Fig 4D). There were no significant differences observed between the groups neither in CCLV-RIE270 nor in POEC. However, in the 200 ng E2/ml group, regions with several successive Ki67 positive cells were frequently observed in POEC (Fig 4C).

### E2 regulates expression of genes related to inflammation and DDR

**Fig 5:**
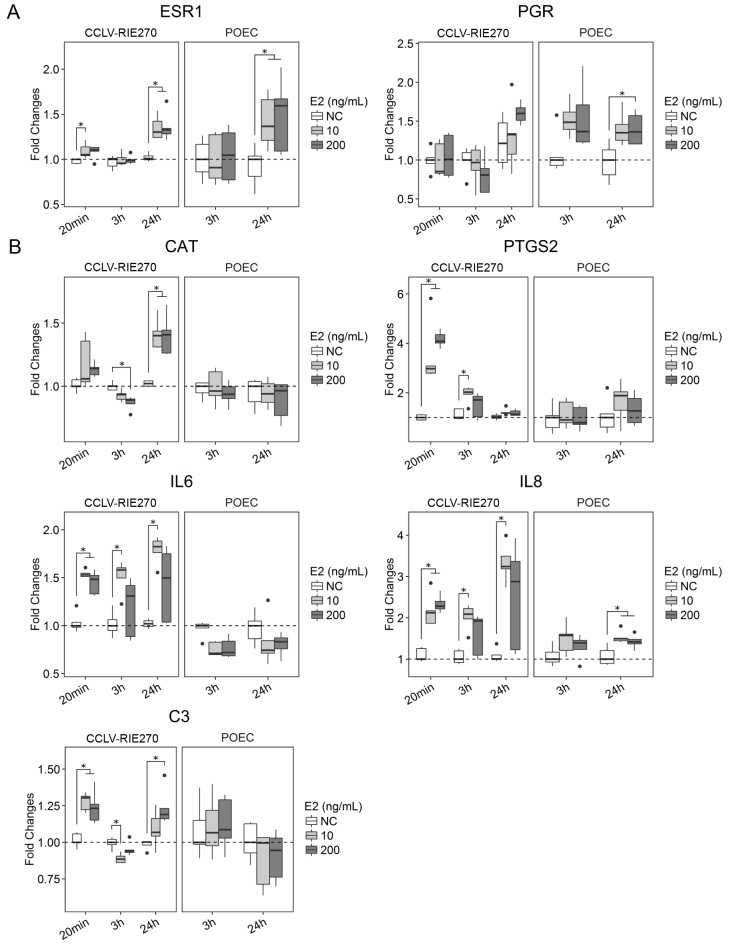
Expression of genes related to E2 activity and inflammatory response in CCLV-RIE270 and POEC in response to stimulation with periovulatory follicular fluid concentrations of E2. A: Expression of steroid receptors (ESR1 and PGR). B: Expression of inflammation-related marker genes. Data is shown as fold changes relative to the control group and normalized with the reference genes *SDHA* and *ACTB*. Asterisks indicate statistical significance (p ≤ 0.05). N = 5 replicates. NC, negative control.

**Fig 6:**
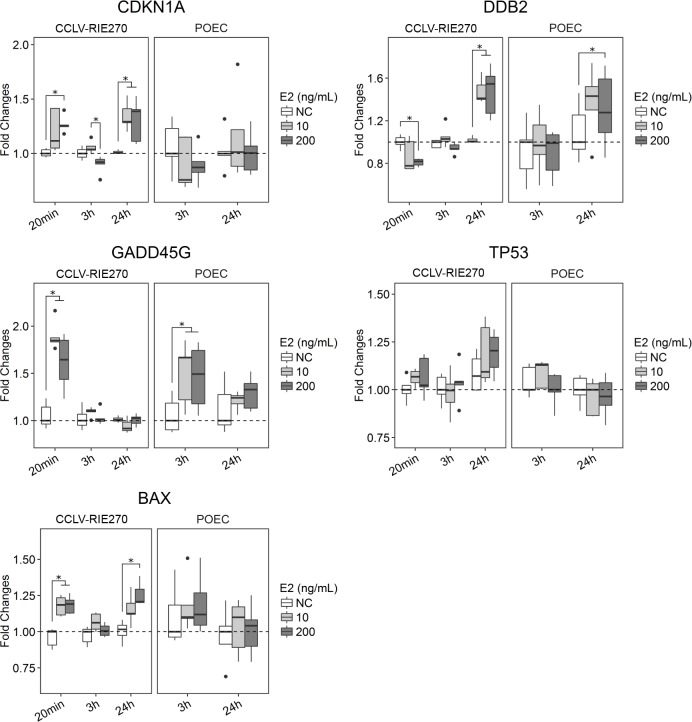
Expression of genes related to DDR in CCLV-RIE270 and POEC in response to stimulation with periovulatory follicular fluid concentrations of E2. Data is shown as fold changes relative to the control group and normalized with the reference genes *SDHA* and *ACTB*. Asterisks indicate statistical significance (p ≤ 0.05). N = 5 replicates. NC, negative control.

Gene expression assessed in this study represented three main aspects of cell response: specific response to E2 (*ESR1*, *PGR*), inflammation (*PTGS2*, *CAT*, *IL8*, *IL6* and *C3*) and DDR (*CDKN1A*, *DDB2*, *GADD45G*, *TP53* and *BAX*). Stimulation with E2 (10 ng/ml and 200 ng/ml) significantly increased the expression of *ESR1* after 24 h in both CCLV-RIE270 and POEC (Fig 5A). PGR was significantly upregulated in the differentiated POEC *in vitro* model only (24 h, 200 ng/ml E2) (Fig 5A). E2 upregulated the expression of *PTGS2* in CCLV-RIE270 in a time and dose dependent manner (Fig 5B). After 24 h, *PTGS2* expression was back to control levels in all groups. Also, *CAT*, *IL8* and *IL6* expression was increased by E2 stimulation in CCLV-RIE270, while *C3* was only moderately affected. POEC only showed slight induction of *IL8* transcription after 24 h of E2 exposure (Fig 5B). In the POEC model none of the E2 concentrations influenced *PTGS2*, *IL6* or *C3* transcription levels at any time point (Fig 5B). Both E2 doses caused an increase in mRNA of *CDKN1A* and *DDB2* after 24 h of E2 exposure in CCLV-RIE270 (Fig 6). In POEC, only expression of *DDB2* was slightly elevated after 24h. *GADD45G* expression was upregulated after 20 min in CCLV-RIE270 and after 3 h in POEC, whereas *TP53* was not affected in any of the culture models (Fig 6). Finally, the expression of the apoptotic gene *BAX* was slightly increased in response to E2 after 20 min and after 24 h (200 ng/ml) in CCLV-RIE270, whereas in POEC its transcription was not affected (Fig 6).

## Discussion

Follicular fluid reaches the distal oviduct epithelium shortly after ovulation and induces an inflammatory-like and DNA damage response. E2 concentrations peak before ovulation in the follicular fluid, reaching much higher levels than in serum [7]. To better understand the role of E2 in the response of the oviduct epithelium to follicular fluid, we evaluated the consequences of follicular fluid E2 on two porcine oviductal epithelial cell models. We hypothesized that apical application of periovulatory follicular levels of E2 would simultaneously trigger inflammatory and DNA damage responses, as well as proliferation.

The exposure of oviduct epithelial cells to follicular fluid concentrations of E2 promoted transcription of *IL6*, *IL8* and *PTGS2*, indicative of an inflammatory-like response. This is in line with results produced by exposing bovine [22] or human [23] oviductal epithelial cells *in vitro* to human follicular fluid. Eddie et al [8] reported an upregulation of IL8 in response to a lower concentration of E2 (10 nM) in oviduct epithelial cells. Similarly, superovulation in mouse promoted the recruitment of pro-inflammatory macrophages in the oviduct [6]. Taken together, this suggests that E2 is at least one of the contributors to the ovulation-related inflammatory response. A possible mechanism for this E2 effect is the production of intracellular ROS, which can induce PTGS2, cytokine and chemokine synthesis [24]. Catalase is an antioxidant enzyme, which fights free radicals, and its level relates to the stage of cellular oxidative stress [25]. After E2 stimulation, expression of catalase was up-regulated in oviductal epithelial cells, reflecting an increased oxidative status. Thus, we hypothesize that at periovulatory follicular levels, E2 could promote an inflammation-like reaction by increasing the levels of ROS in oviduct epithelial cells.

The proliferative role of E2 has been reported in other cell types [9,10], however, after determination of impedance and Ki67 expression, 24 h after applying E2 at follicular fluid levels, no significant induction of proliferation was induced in our oviduct epithelial cell models. This is in line with a previous *in vitro* study in human fallopian tube cells, where exposure to E2 for 7 days did not induce proliferation [8].

Upregulation of *CDKN1A*, *DDB2* and *GADD45G* indicated that DDR was triggered by high-level E2 stimulation. After DNA damage, transcription of *CDKN1A*, *DDB2* and *GADD45G* is induced by activation of p53, a transcription factor encoded by *TP53* [26,27]. Transcription of *TP53* was not significantly affected by the E2 treatment, but the upregulation of its downstream effectors indicates that the follicular fluid E2 induces DDR in oviduct epithelial cells.

Discrepancies in gene expression were found between the two models. There are two key distinctions that might explain why the pronounced inflammatory and DNA damage responses to E2 seen in CCLV-RIE270 were not recapitulated by POEC. First, secretory oviduct epithelial cells are more prone to genotoxic stress than ciliated cells [28]. Thus, if the same genotoxic stimulus is applied, milder responses will be triggered in cultures having both ciliated and secretory cell populations (e.g. POEC), in contrast to cultures consisting of pure secretory cells (e.g. CCLV-RIE270). Second, there are structural differences between CCLV-RIE270 and POEC. For instance, the surface of exposure to E2 is larger in CCLV-RIE270 (non-polarized, flat cells) than in POEC (polarized, columnar shaped cells). Other differences include specific cell to cell contacts and membrane properties.

Considering that spontaneous serous ovarian cancer is linked to increased number of lifetime ovulations in humans [6], and that it is likely to arise from the oviductal tube epithelium [29], the long term cumulative effects of follicular fluid components on oviduct epithelial cells deserve special attention. Moreover, porcine models for the study of human diseases hold great potential [12,13], which is why we consider POEC and CCLV-RIE270 as suitable *in vitro* models to understand the early origins of serous ovarian cancer in the oviduct. On the one hand, POEC can be maintained *in vitro* in a highly differentiated state [14–16] without passaging for at least 3 months [30], offering the possibility to evaluate the effects of follicular fluid components repeatedly over several weeks on a mixture of secretory and ciliated cells. On the other hand, CCLV-RIE270 is immortalized, easy to use, and provides a pure secretory cell population, preserving their original phenotype, as shown by expression of specific marker proteins (especially OVGP1, a functional marker for oviductal secretory cells) and by the absence of anchorage independent growth.

We conclude that CCLV-RIE270 and POEC can be used as complementary *in vitro* systems to evaluate the consequences of oviductal exposure to follicular fluid components. Using these two models we were able to demonstrate that single administration of E2 at periovulatory follicular fluid levels simultaneously activates DDR and inflammation, but not proliferation in oviduct epithelial cells *in vitro*.
